# Supplementary figures and images for: Multiangle perception-oriented environmental facility design method based on joint fuzzy decision-making and transfer learning
Source: PeerJ Comput Sci. 2024 Mar 28;10:e1855. doi: 10.7717/peerj-cs.1855 (PMC11041929; doi:10.7717/peerj-cs.1855)

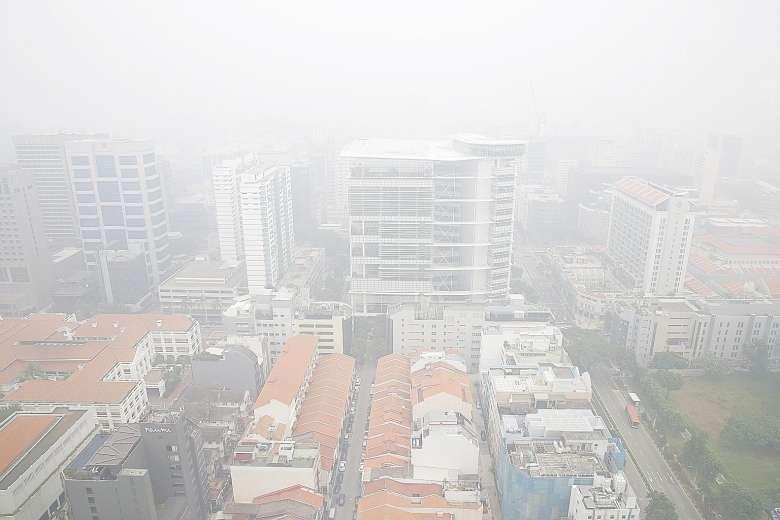

Supplement: Supplemental Information 2 [file peerj-cs-10-1855-s002.zip › Data-0217/Data_2/1.jpg]

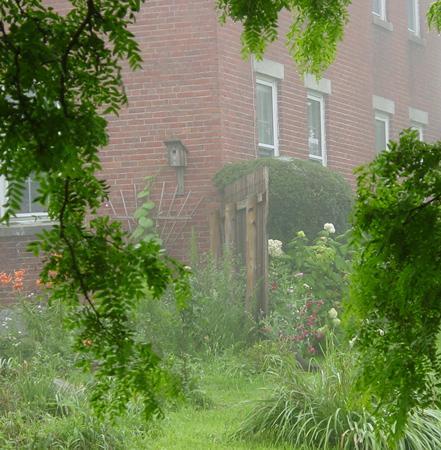

Supplement: Supplemental Information 2 [file peerj-cs-10-1855-s002.zip › Data-0217/Data_2/10.jpg]

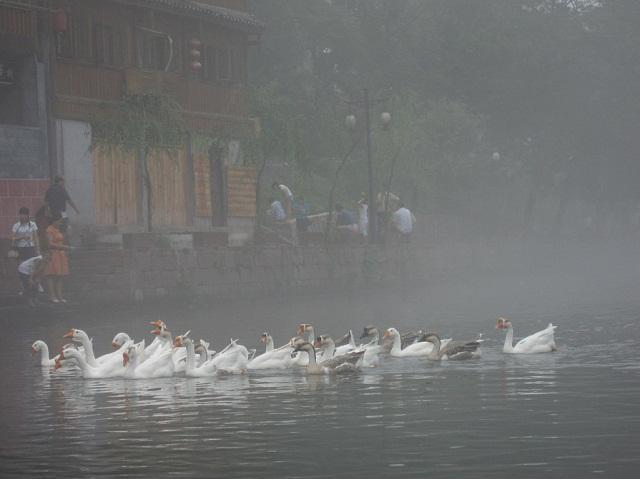

Supplement: Supplemental Information 2 [file peerj-cs-10-1855-s002.zip › Data-0217/Data_2/11.jpg]

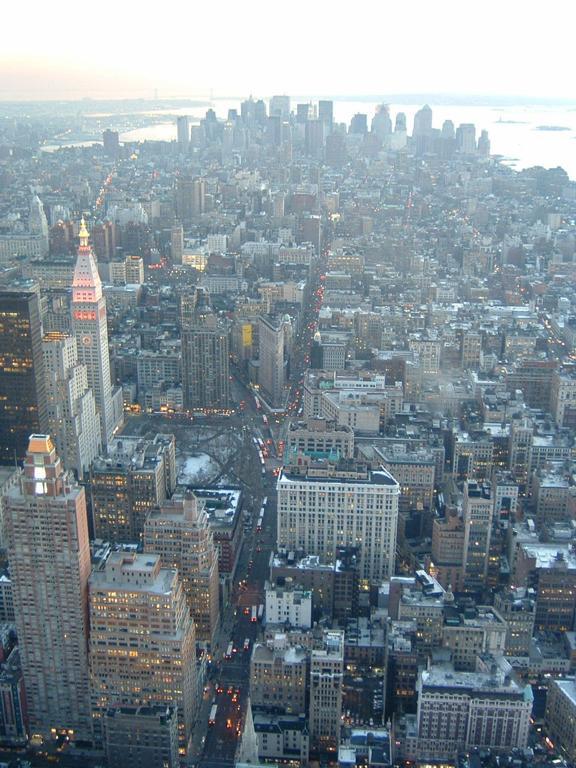

Supplement: Supplemental Information 2 [file peerj-cs-10-1855-s002.zip › Data-0217/Data_2/12.jpg]

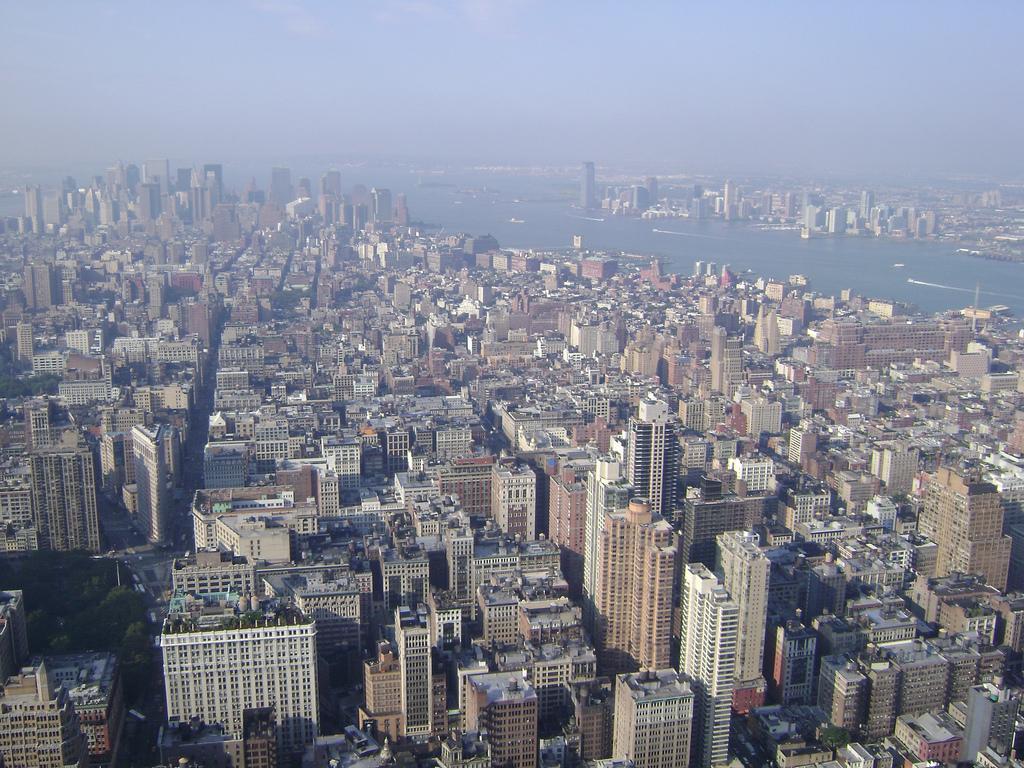

Supplement: Supplemental Information 2 [file peerj-cs-10-1855-s002.zip › Data-0217/Data_2/13.jpg]

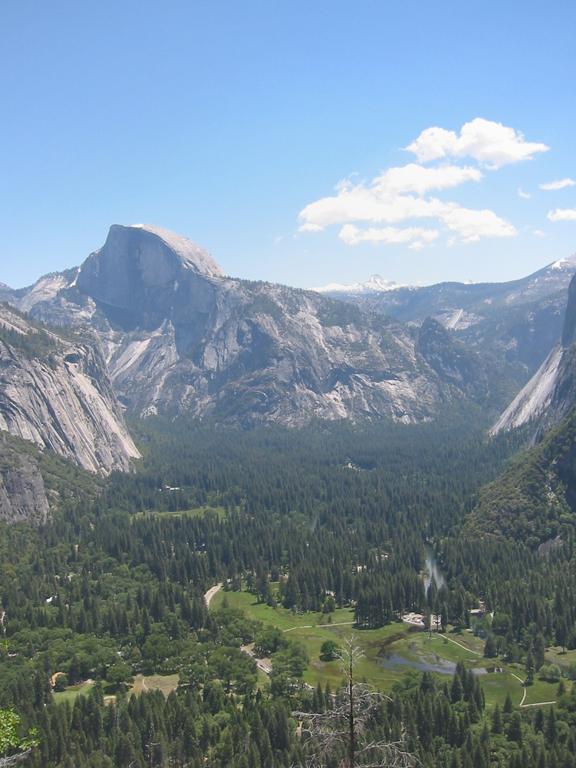

Supplement: Supplemental Information 2 [file peerj-cs-10-1855-s002.zip › Data-0217/Data_2/14.jpg]

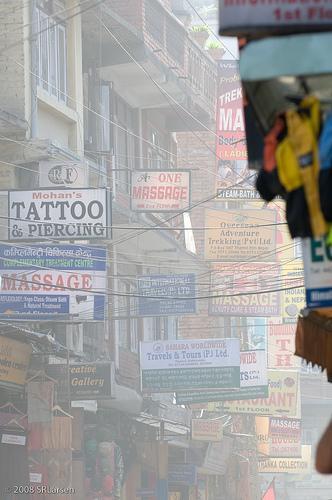

Supplement: Supplemental Information 2 [file peerj-cs-10-1855-s002.zip › Data-0217/Data_2/15.jpg]

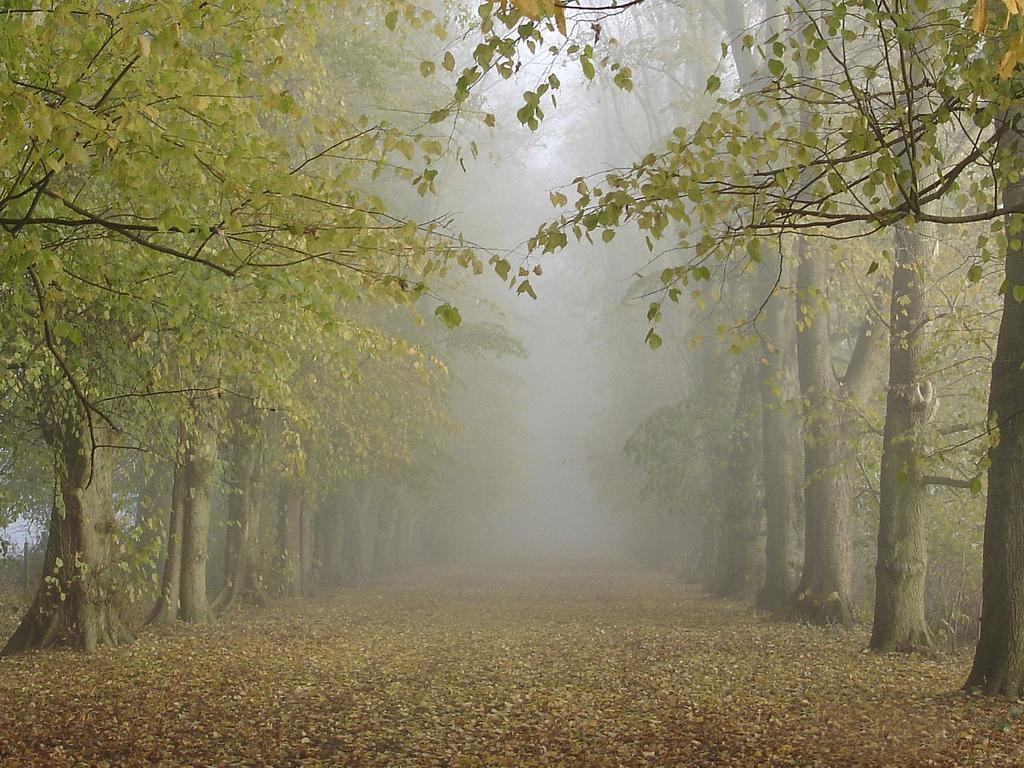

Supplement: Supplemental Information 2 [file peerj-cs-10-1855-s002.zip › Data-0217/Data_2/16.jpg]

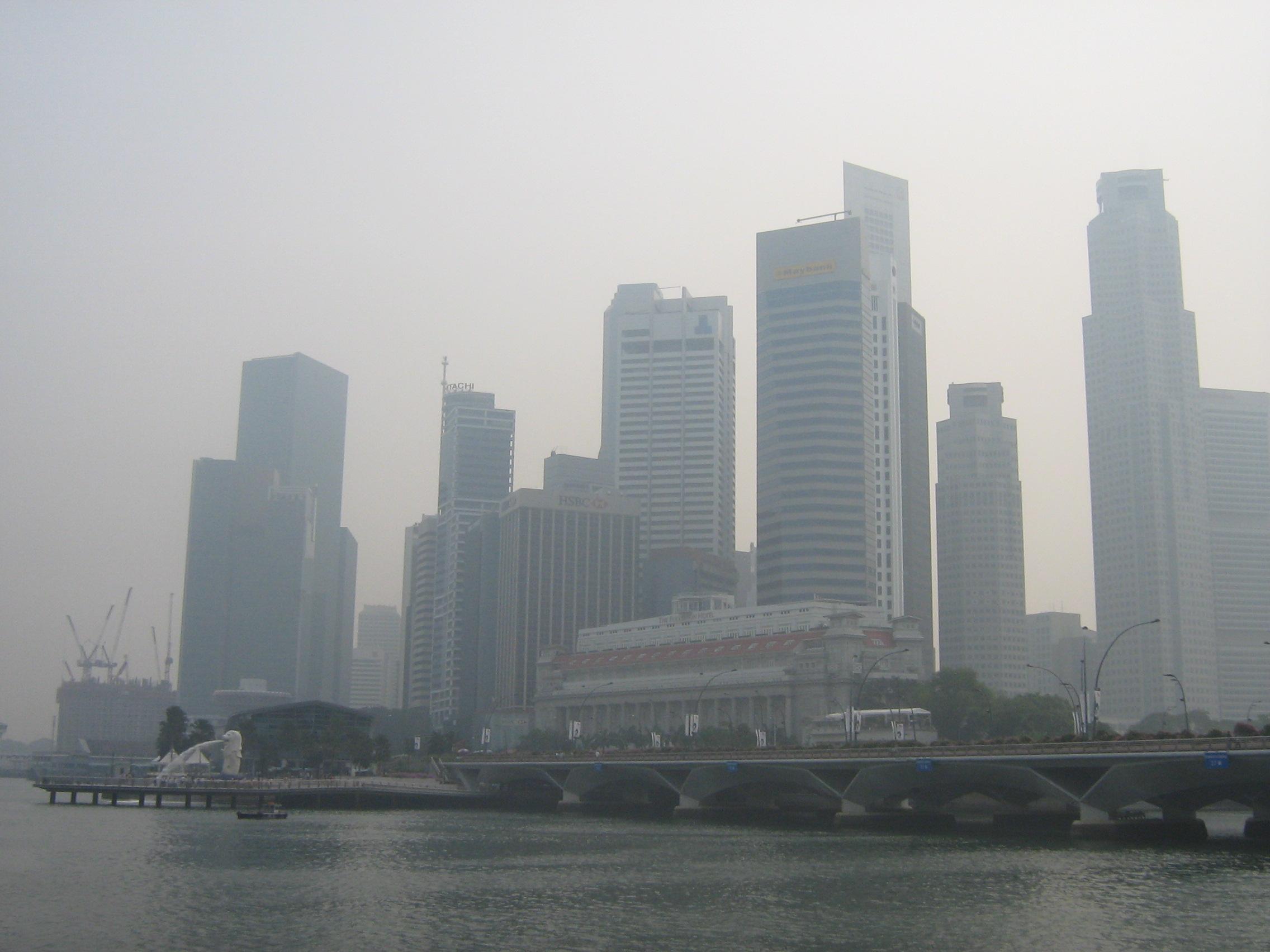

Supplement: Supplemental Information 2 [file peerj-cs-10-1855-s002.zip › Data-0217/Data_2/17.jpg]

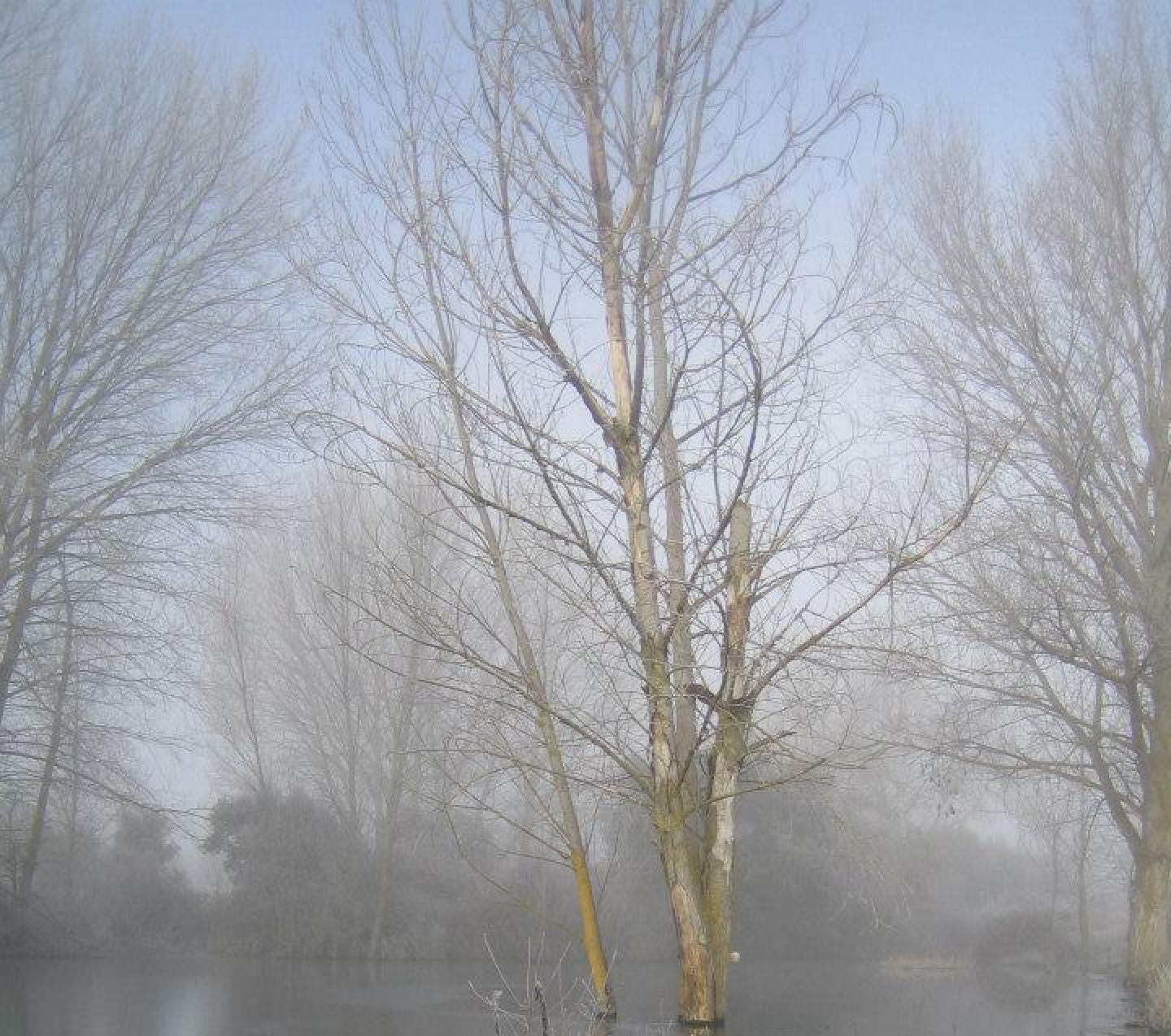

Supplement: Supplemental Information 2 [file peerj-cs-10-1855-s002.zip › Data-0217/Data_2/18.jpg]

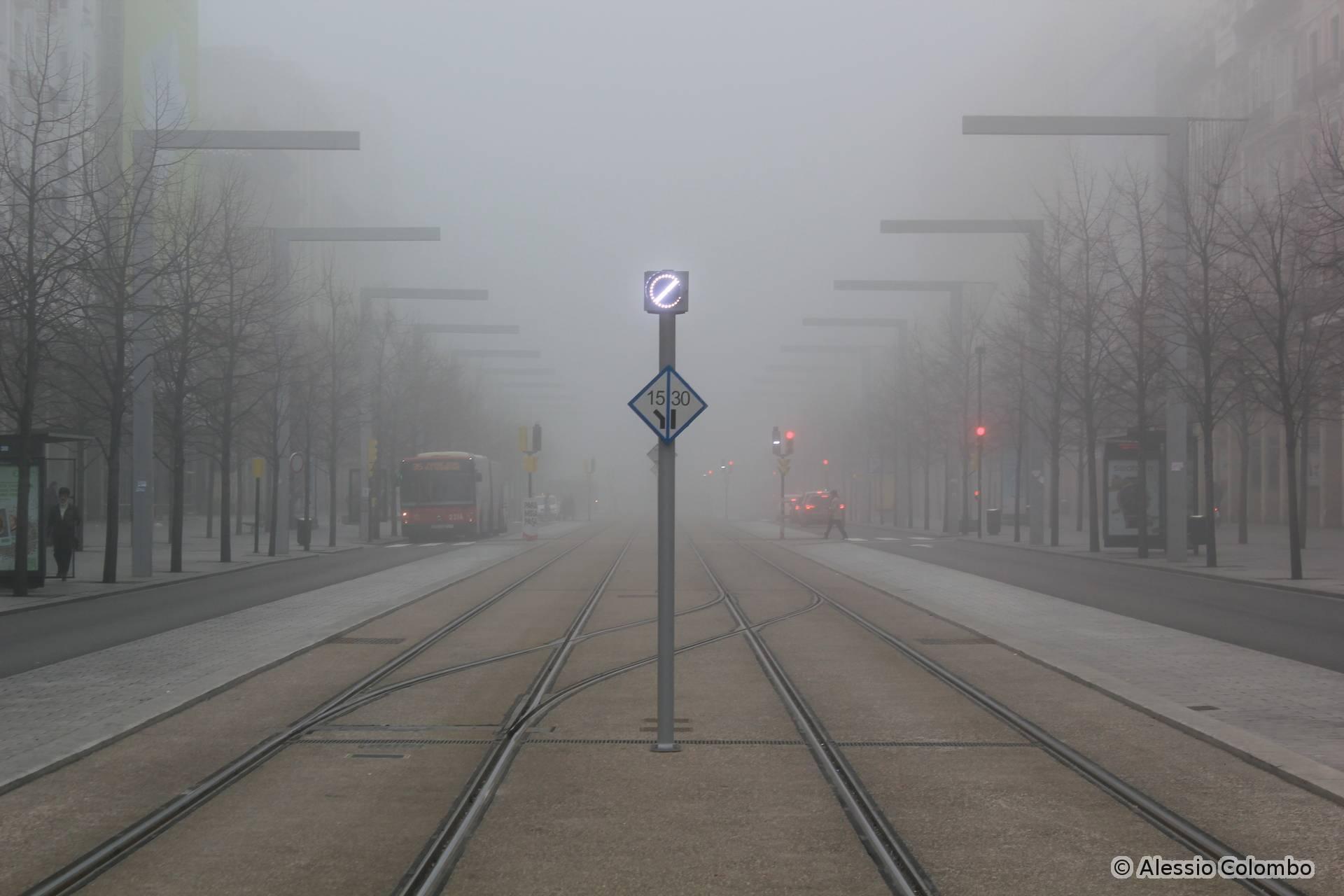

Supplement: Supplemental Information 2 [file peerj-cs-10-1855-s002.zip › Data-0217/Data_2/19.jpg]

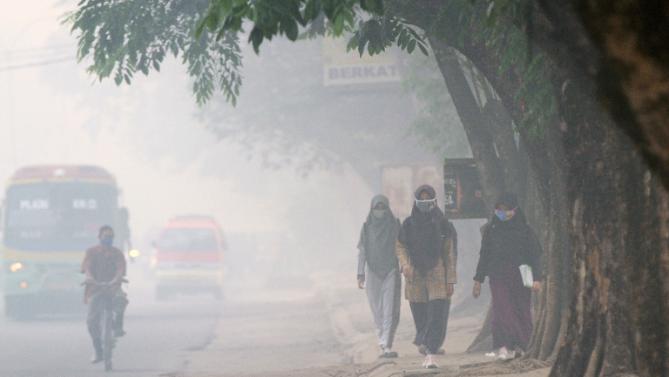

Supplement: Supplemental Information 2 [file peerj-cs-10-1855-s002.zip › Data-0217/Data_2/2.jpg]

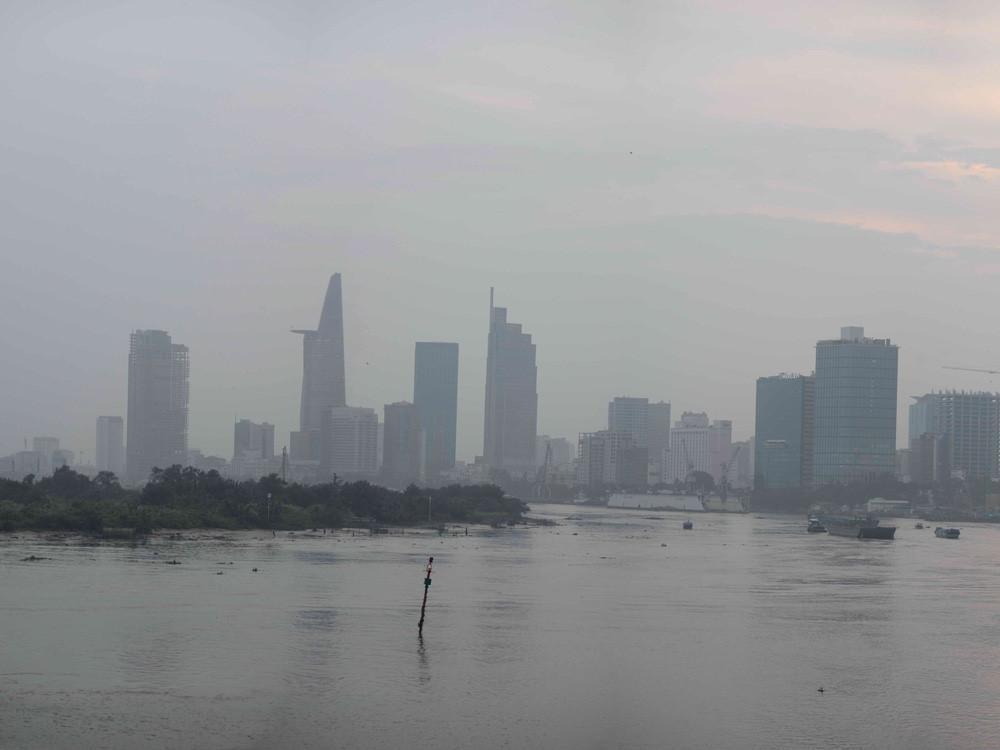

Supplement: Supplemental Information 2 [file peerj-cs-10-1855-s002.zip › Data-0217/Data_2/20.jpg]

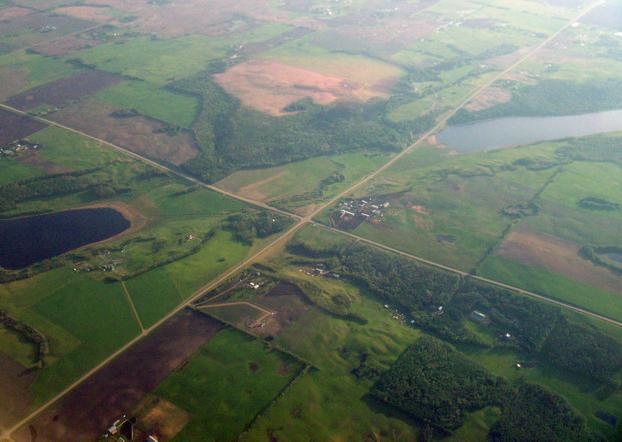

Supplement: Supplemental Information 2 [file peerj-cs-10-1855-s002.zip › Data-0217/Data_2/21.jpg]

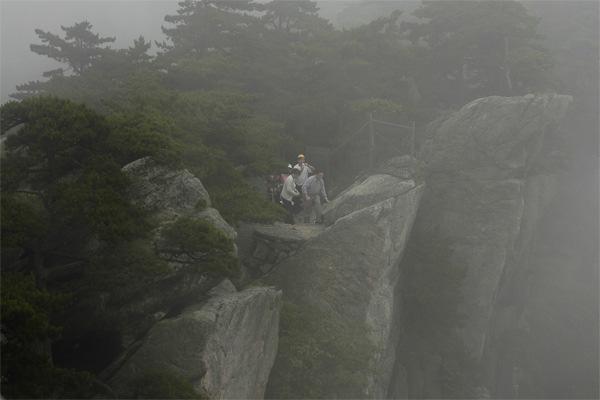

Supplement: Supplemental Information 2 [file peerj-cs-10-1855-s002.zip › Data-0217/Data_2/22.jpg]

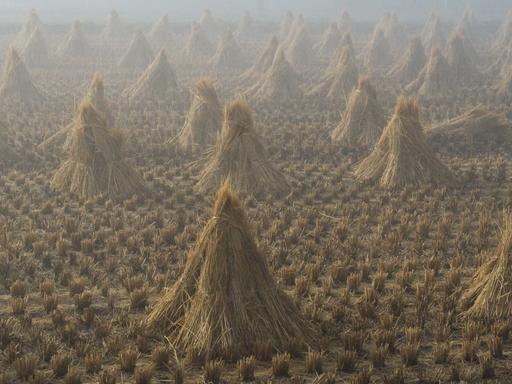

Supplement: Supplemental Information 2 [file peerj-cs-10-1855-s002.zip › Data-0217/Data_2/23.jpg]

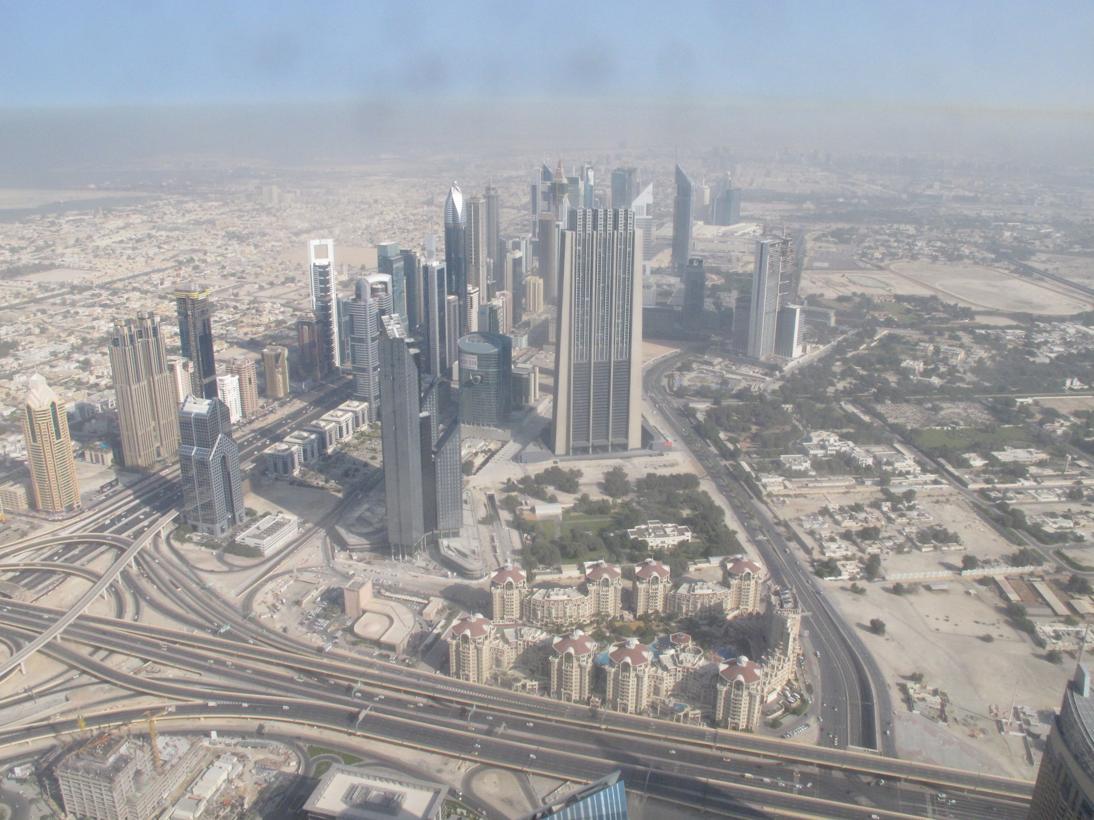

Supplement: Supplemental Information 2 [file peerj-cs-10-1855-s002.zip › Data-0217/Data_2/24.jpg]

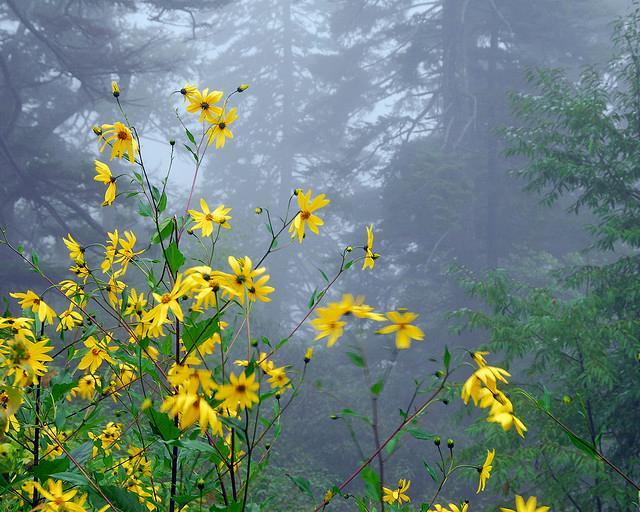

Supplement: Supplemental Information 2 [file peerj-cs-10-1855-s002.zip › Data-0217/Data_2/25.jpg]

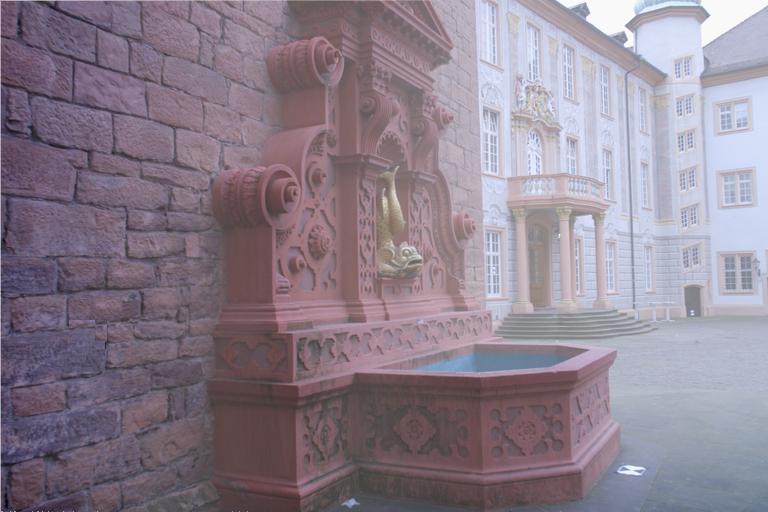

Supplement: Supplemental Information 2 [file peerj-cs-10-1855-s002.zip › Data-0217/Data_2/26.jpg]

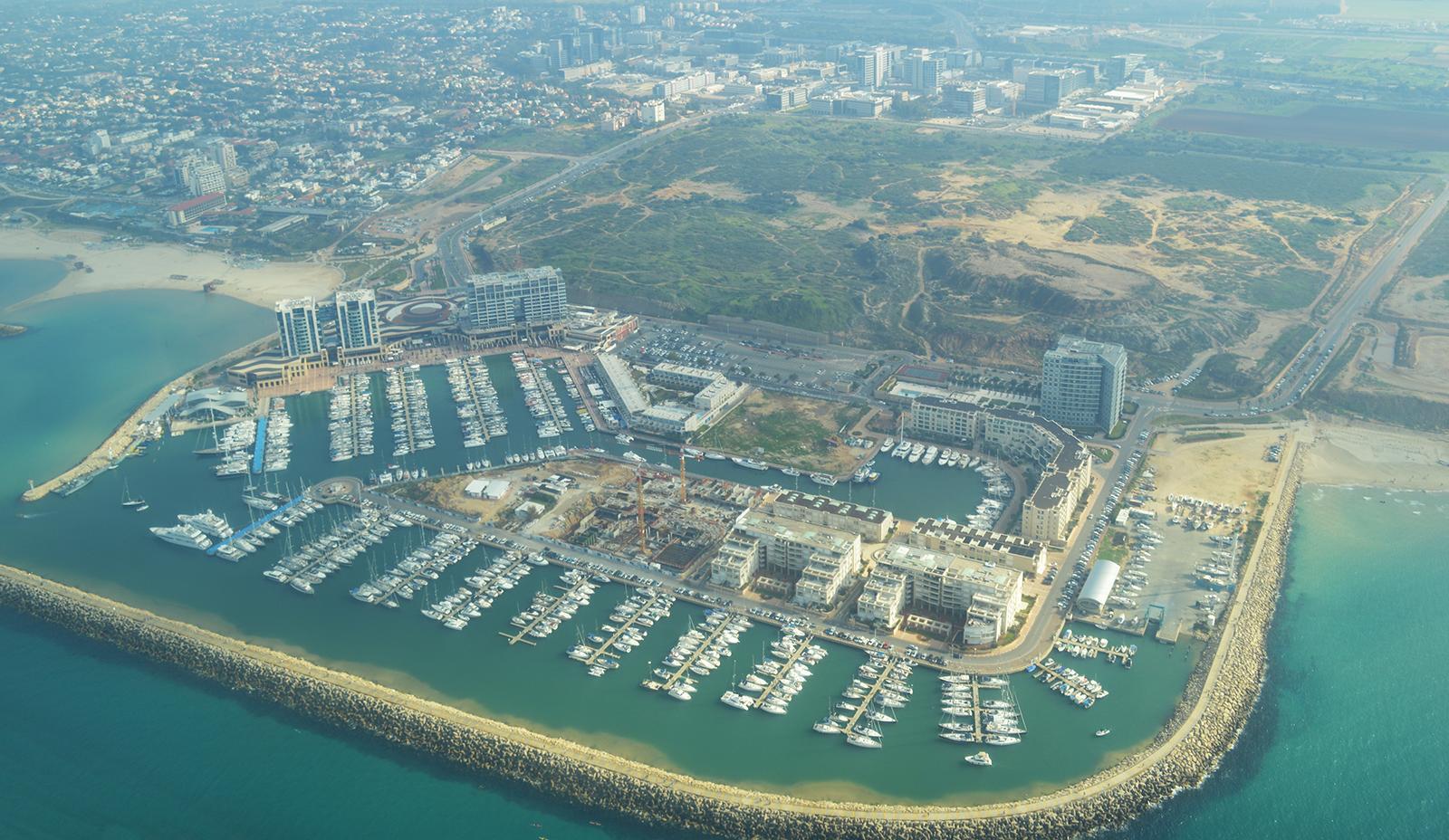

Supplement: Supplemental Information 2 [file peerj-cs-10-1855-s002.zip › Data-0217/Data_2/27.jpg]

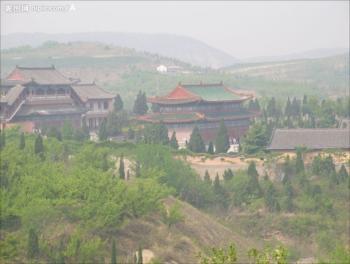

Supplement: Supplemental Information 2 [file peerj-cs-10-1855-s002.zip › Data-0217/Data_2/28.jpg]

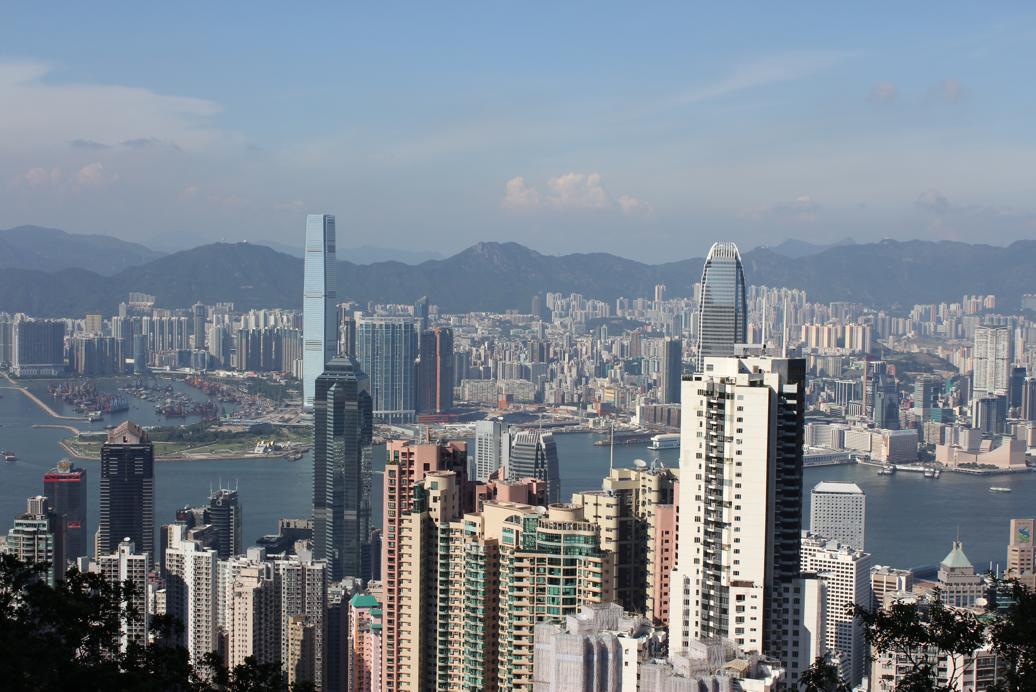

Supplement: Supplemental Information 2 [file peerj-cs-10-1855-s002.zip › Data-0217/Data_2/29.jpg]

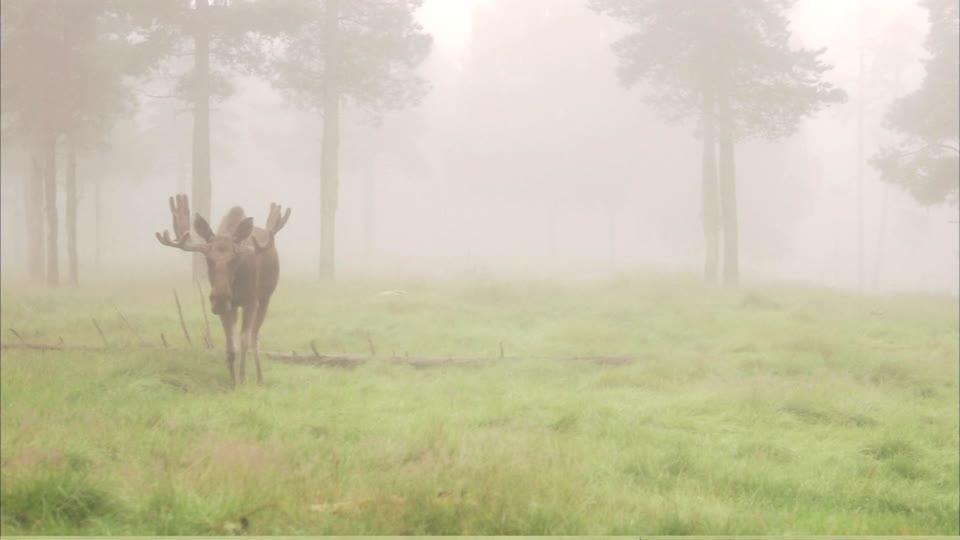

Supplement: Supplemental Information 2 [file peerj-cs-10-1855-s002.zip › Data-0217/Data_2/3.jpg]

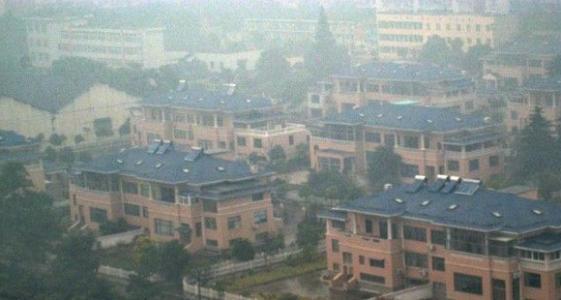

Supplement: Supplemental Information 2 [file peerj-cs-10-1855-s002.zip › Data-0217/Data_2/30.jpg]

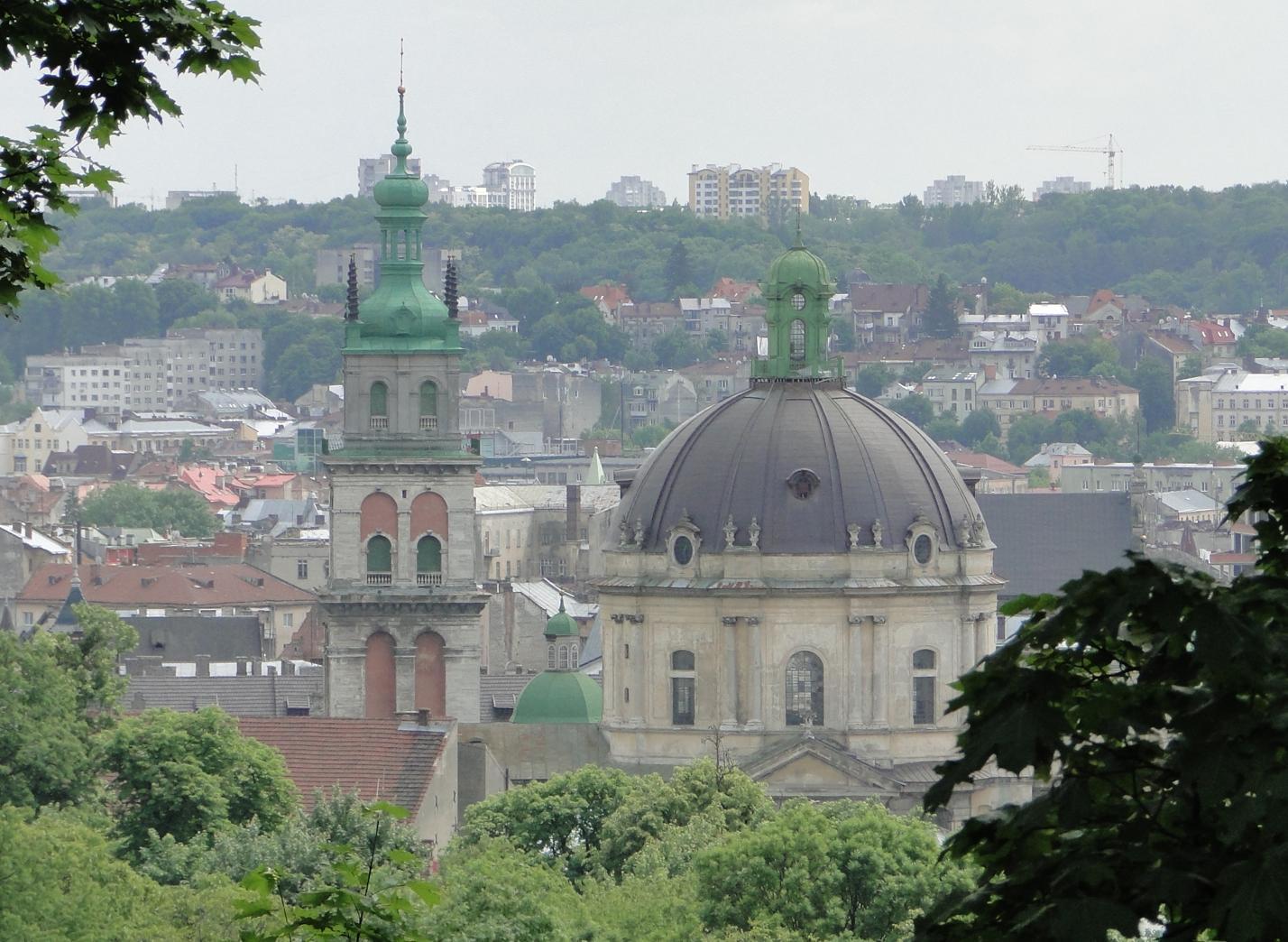

Supplement: Supplemental Information 2 [file peerj-cs-10-1855-s002.zip › Data-0217/Data_2/31.jpg]

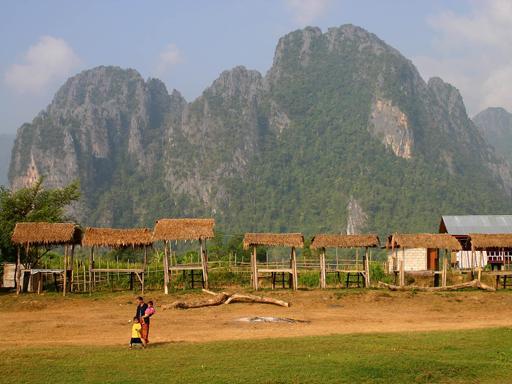

Supplement: Supplemental Information 2 [file peerj-cs-10-1855-s002.zip › Data-0217/Data_2/32.jpg]

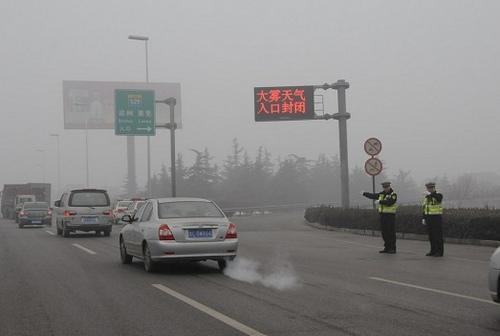

Supplement: Supplemental Information 2 [file peerj-cs-10-1855-s002.zip › Data-0217/Data_2/33.jpg]

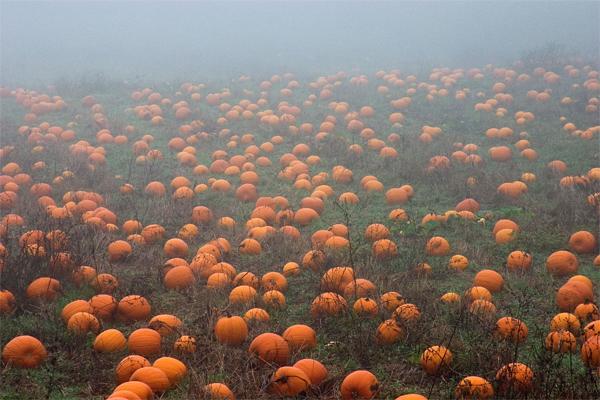

Supplement: Supplemental Information 2 [file peerj-cs-10-1855-s002.zip › Data-0217/Data_2/34.jpg]

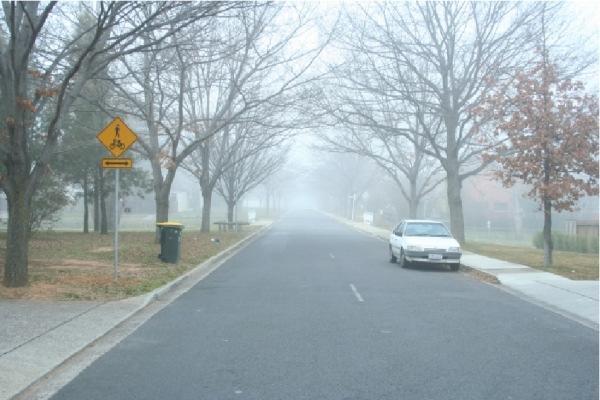

Supplement: Supplemental Information 2 [file peerj-cs-10-1855-s002.zip › Data-0217/Data_2/35.jpg]

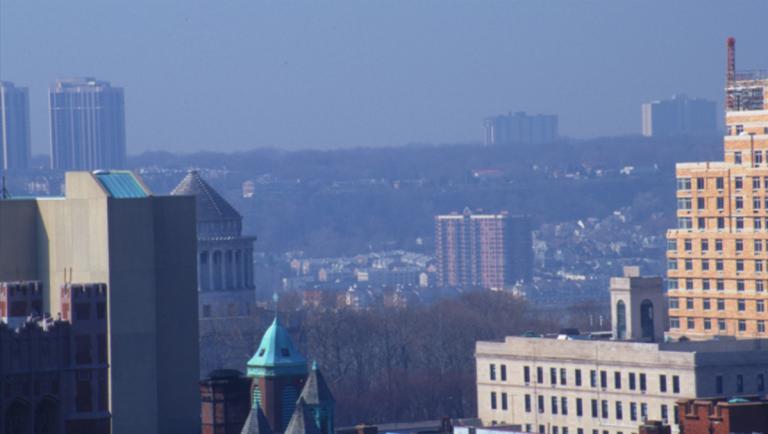

Supplement: Supplemental Information 2 [file peerj-cs-10-1855-s002.zip › Data-0217/Data_2/36.jpg]

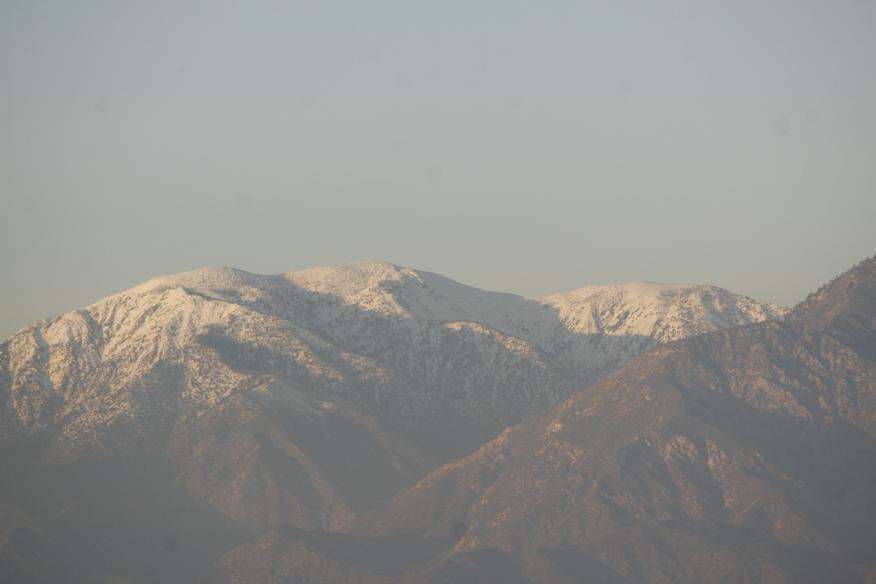

Supplement: Supplemental Information 2 [file peerj-cs-10-1855-s002.zip › Data-0217/Data_2/37.jpg]

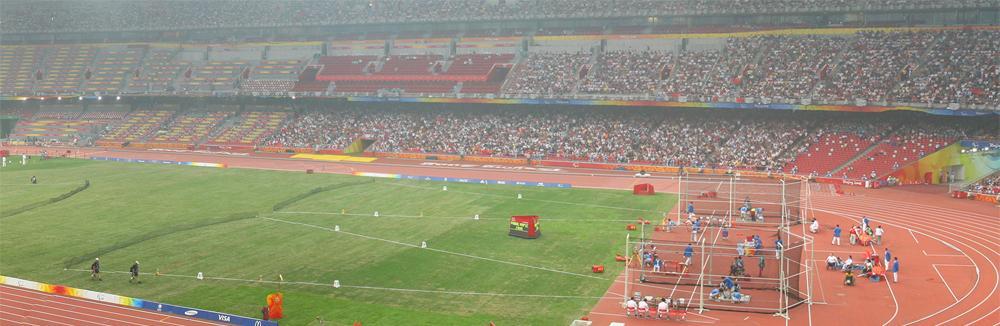

Supplement: Supplemental Information 2 [file peerj-cs-10-1855-s002.zip › Data-0217/Data_2/38.jpg]

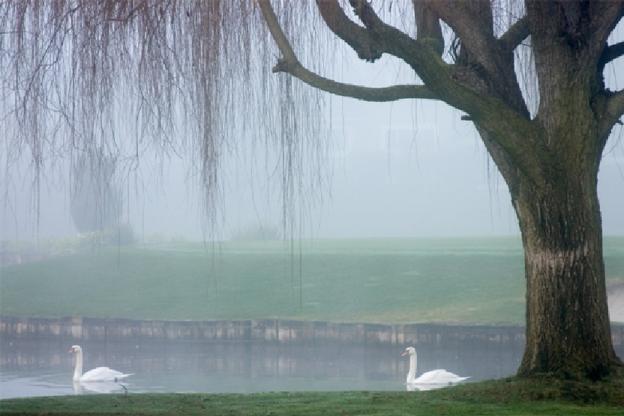

Supplement: Supplemental Information 2 [file peerj-cs-10-1855-s002.zip › Data-0217/Data_2/39.jpg]

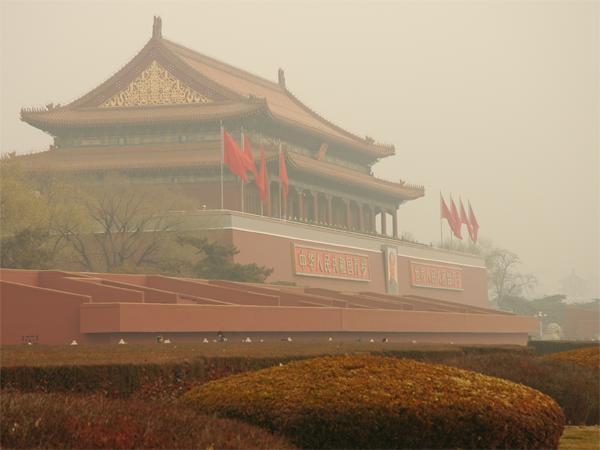

Supplement: Supplemental Information 2 [file peerj-cs-10-1855-s002.zip › Data-0217/Data_2/4.jpg]

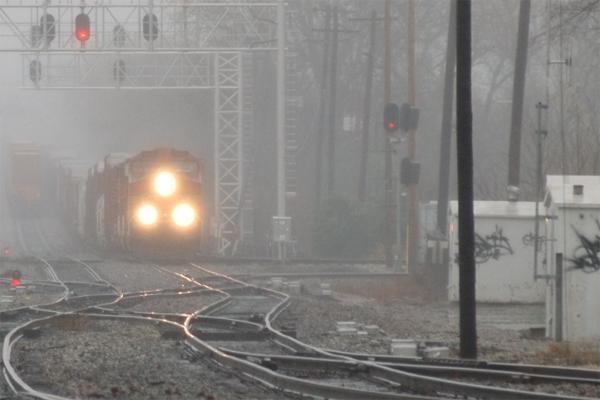

Supplement: Supplemental Information 2 [file peerj-cs-10-1855-s002.zip › Data-0217/Data_2/40.jpg]

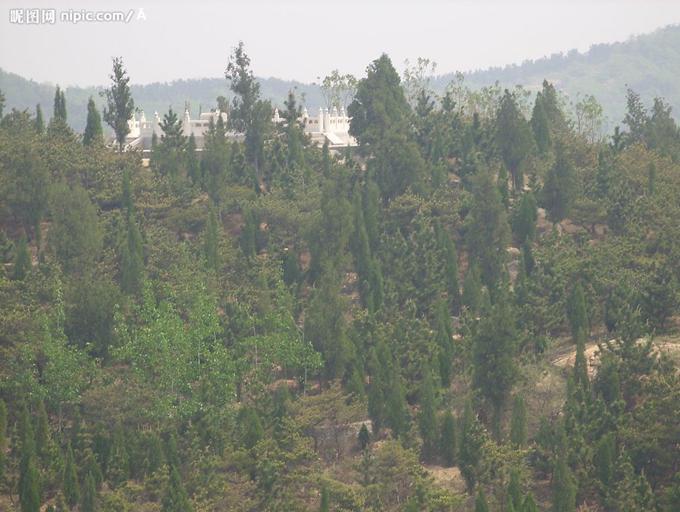

Supplement: Supplemental Information 2 [file peerj-cs-10-1855-s002.zip › Data-0217/Data_2/41.jpg]

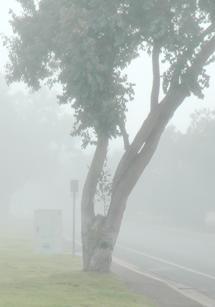

Supplement: Supplemental Information 2 [file peerj-cs-10-1855-s002.zip › Data-0217/Data_2/42.jpg]

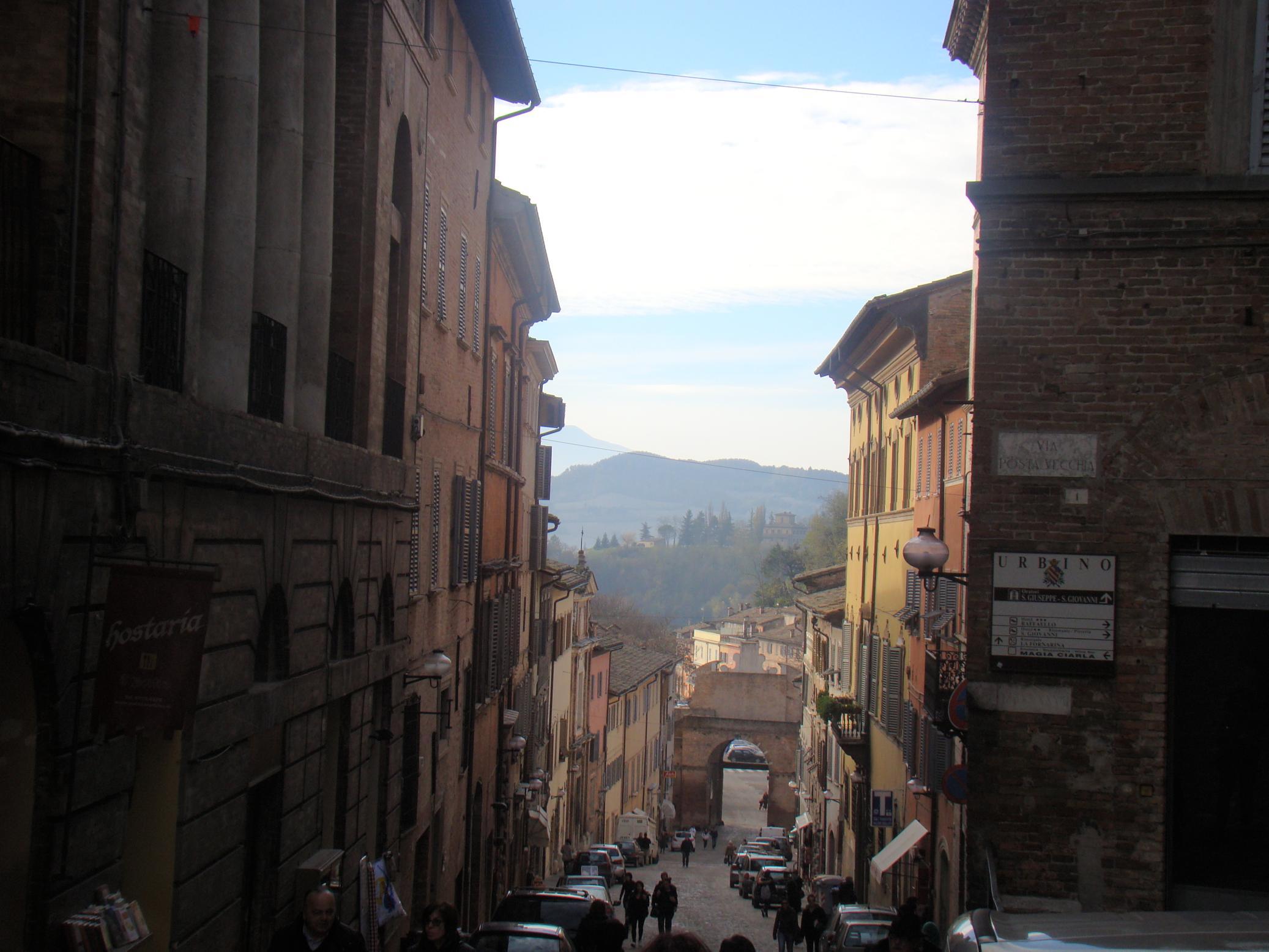

Supplement: Supplemental Information 2 [file peerj-cs-10-1855-s002.zip › Data-0217/Data_2/43.jpg]

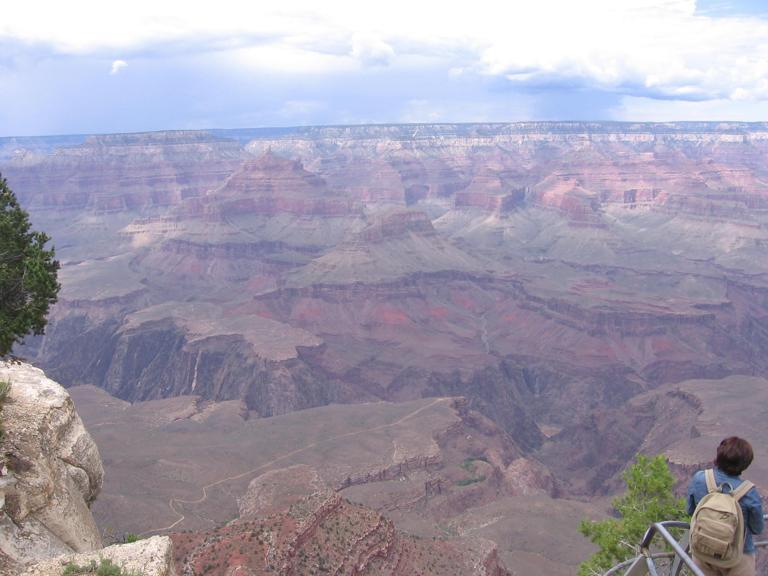

Supplement: Supplemental Information 2 [file peerj-cs-10-1855-s002.zip › Data-0217/Data_2/5.jpg]

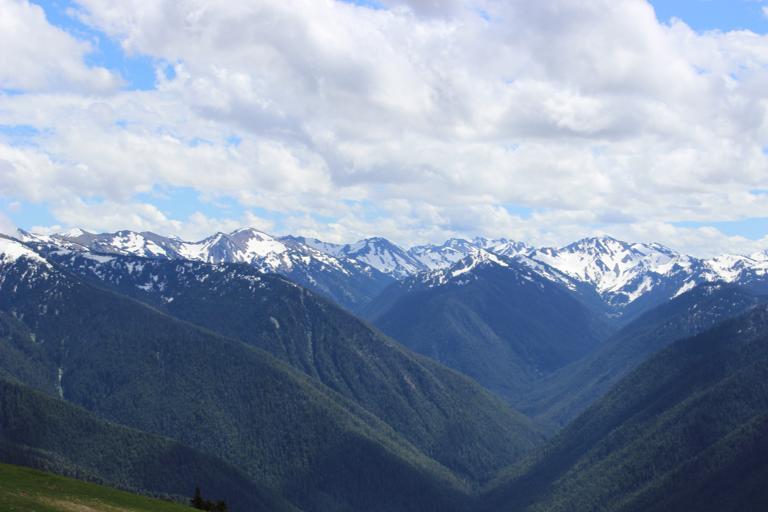

Supplement: Supplemental Information 2 [file peerj-cs-10-1855-s002.zip › Data-0217/Data_2/9.jpg]
