# Supplementary material for: Multiangle perception-oriented environmental facility design method based on joint fuzzy decision-making and transfer learning
Source: PeerJ Comput Sci. 2024 Mar 28;10:e1855. doi: 10.7717/peerj-cs.1855 (PMC11041929; doi:10.7717/peerj-cs.1855)
Supplement: Supplemental Information 3 [file peerj-cs-10-1855-s003.docx]

**Market survey questionnaire for seats**

1. Your gender:

□ male

□ female

2. What is your age?

□ Under 15 years old

□ 16-25 years old

□ 26 to 35 years old

□ 36-45 years old

□ Over 45 years old

3. What is your profession?

□ student

□ Unit employees

□ administrative staff

□ Freelancing

□ other

4. What is your favorite chair shape?

□ rotundity

□ square

□ triangle

□ Irregular shape

5. Are you dissatisfied with the current home chairs?

□ Stupid design

□ Large footprint

□ Not easy to clean

□ Not comfortable

□ Lack of functionality

□ other

6. What do you think are the standards for a good chair?

□ Quality compliance

□ Environmental protection of materials used

□ Good looking design

□ Low price

□ Comfortable to use

□ other

7. What is your favorite chair color?

□ monochrome

□ Flower color

8. What style of chair do you want to purchase?

□ Modern minimalism

□ Mediterranean style

□ american style

□ Japanese Style

□ european style

□ Rural style

□ Mix and match style

□ other

9. How did you learn about this product?

□ network

□ Introduction from friends/classmates

□ Furniture store

□ other

10. What is the maximum acceptable price range for this product for you?

□ Below 500

□ 500~1500

□ 1500~3000

□ 3000~6000

□ Over 6000

11. What is the most attractive way to attract you to purchase the product (chair)?

□ Buy a few and get a few free

□ Discounts

□ Reward promotion

□ Giving gifts

□ other

12. If you are willing to purchase, where would you purchase it?

□ furniture centre

□ Online ordering

□ Supermarkets/hypermarkets

13. What are the key factors that led you to purchase this chair?

□ Good quality

□ Brand awareness

□ The price is reasonable

□ Doing promotions

□ other

14. What do you think are the standards for a good chair?

□ Quality compliance

□ Environmental protection of materials used

□ Good looking design

□ Low price

□ Diverse functions

15. How long do you use your seat every day?

□ Under 30 minutes

□ 30-60 minutes

□ 60-90 minutes

□ Over 90 minutes

16. What is the texture of the chair you like?

□ Soft

□ Harder

□ Moderate softness and hardness

□ other

17. How do you view chairs as versatile?

□ No need

□ It is necessary

□ It doesn't matter

□ other

18. How many chairs did you purchase?

□ 1 unit

□ Two

□ 2 or more

19. What are your main needs for purchasing chairs?

□ Entertain guests

□ Used for office/study

□ Comfortable/aesthetically pleasing to use

□ other

20. What are your requirements for the materials used to make chairs?

□ Leather surface

□ Cloth surface

□ Iron

□ plastic

□ wooden

□ other

21. Do you think the fashion sense of chairs is strong now?

□ There are some fashion elements

□ Foldable variation

□ modelling

□ colour

□ material quality

□ other

22. What is the issue you are most concerned about? What other areas do you think need improvement?
