# Supplementary material for: Multiangle perception-oriented environmental facility design method based on joint fuzzy decision-making and transfer learning
Source: PeerJ Comput Sci. 2024 Mar 28;10:e1855. doi: 10.7717/peerj-cs.1855 (PMC11041929; doi:10.7717/peerj-cs.1855)
Supplement: Supplemental Information 4 [file peerj-cs-10-1855-s004.docx]

**Product Satisfaction Survey Question**

Thank you very much for your strong support. In order to improve our products and services, and increase customer satisfaction, please fill out this survey questionnaire. We will make improvements in our future services. Thank you for your help!

**Note:** Quantitative indicators of satisfaction - very satisfied (90-100 points), satisfied (80-89 points), average (60-79 points), dissatisfied (below 60 points)

| Product | | | | |
| --- | --- | --- | --- | --- |
|  | very satisfied | satisfied | average | dissatisfied |
| Quality |  |  |  |  |
| Stability |  |  |  |  |
| Compatibility |  |  |  |  |
| Visual Effect |  |  |  |  |
| Functionality |  |  |  |  |
| Friendliness |  |  |  |  |
| Influence |  |  |  |  |
| Subjective issues | | | | |
| 1. What do you think is the greatest benefit that the product brings to you? Or what do you think are its advantages? | | | | |
| 2. What do you think is the biggest flaw of the product? Or what areas do you think need improvement? | | | | |
| 3. How many problems have you encountered while using the product? Has the problem been resolved in a timely manner? | | | | |
| 4. Do you have any other needs or requirements for the product? | | | | |
